# The evaluation of motor deficits in children with ulcerative colitis: a cross-sectional study

**DOI:** 10.1007/s00431-026-07014-1

**Published:** 2026-05-04

**Authors:** Ulas Emre Akbulut, Atike Atalay, İshak Abdurrahman Isik, Nazlı Sivil, Memduha Sarı, Gokhan Birsen, Ozgun Kaya Kara, Koray Kara, Hazal Sonbahar Ulu, Hasan Ali İnal

**Affiliations:** 1 Department of Pediatric Gastroenterology Hepatology and Nutrition, University of Health Sciences, Antalya Education and Research Hospital, Varlık Mh. Kazım Karabekir Cd., 07100 Antalya, Turkey; 2 Department of Physiotherapy and Rehabilitation, Akdeniz University, Antalya, Turkey; 3 Department of Child Psychiatry, University of Health Sciences, Antalya Education and Research Hospital, Antalya, Turkey; 4 Department of Obstetrics and Gynecology, University of Health Sciences, Antalya Education and Research Hospital, Antalya, Turkey

**Keywords:** Child, Erythrocyte sedimentation rate, Inflammation, Inflammatory bowel disease, Motor proficiency, Muscle strength

## Abstract

While the gastrointestinal symptoms of ulcerative colitis (UC) are well-defined, its impact on motor development in children remains poorly understood. We aimed to assess motor proficiency, handgrip strength, and physical activity levels in children with UC and to investigate associations with disease activity. In this cross-sectional study, 49 children with UC aged 8–17 years and 41 age‑ and sex-matched healthy controls were enrolled. Disease activity was assessed using the Pediatric Ulcerative Colitis Activity Index, and laboratory markers including C-reactive protein and erythrocyte sedimentation rate were measured. Motor proficiency was evaluated using the Bruininks-Oseretsky Test of Motor Proficiency, Second Edition. Handgrip strength was measured with a dynamometer, and physical activity was assessed via the Physical Activity Questionnaire (PAQ). Group comparisons were adjusted for age, sex, socioeconomic status, and body mass index, with False Discovery Rate correction for multiple testing. Children with UC scored significantly lower on the Total Motor Composite than controls (37.4 vs. 45.1, *p* < 0.001). Adjusted analyses revealed a specific pattern: fine motor skills were preserved, but significant deficits were found in gross motor domains, including bilateral coordination and strength and agility (all *p* < 0.001). Handgrip strength differences lost significance after adjustment for body mass index, and no difference was observed in physical activity. Within the UC group, a higher erythrocyte sedimentation rate correlated with lower non-dominant handgrip strength (*ρ* =  − 0.533, *p* < 0.001) and poorer agility (*ρ* =  − 0.414, *p* = 0.003).

*Conclusion*: Children with UC exhibit significant gross motor deficits, even during clinical remission. These cross-sectional findings indicate that clinically well children may still have impaired motor proficiency compared to healthy peers. However, longitudinal studies are needed to determine whether these deficits predate diagnosis, emerge during active disease, or persist despite remission.

**Table:** 

**What is Known:** • *The primary treatment goal in ulcerative colitis with children is the resolution of symptoms and intestinal inflammation.* • *The potential effects of ulcerative colitis on a child’s motor function are not routinely assessed in clinical practice.*	
**What is New:** • *Children with ulcerative colitis have significantly lower overall motor proficiency scores compared to their healthy peers.* • *Children in clinical remission may still have impaired motor skills, indicating a potential need for comprehensive functional assessment during follow-up.*

**Supplementary Information:**

The online version contains supplementary material available at 10.1007/s00431-026-07014-1.

## Introduction

Ulcerative colitis (UC) is a chronic, immune-mediated inflammatory bowel disease (IBD) characterized by continuous mucosal inflammation extending proximally from the rectum [1]. Pediatric-onset UC represents a distinct phenotype that is frequently associated with extensive colonic involvement, aggressive disease course, and nutritional compromise during critical developmental years [2, 3].

Children with UC face a debilitating cycle that impairs physical health. Persistent inflammation, elevated pro-inflammatory cytokines, and malabsorption cause deficiencies in essential nutrients like calcium, zinc, and vitamin D [4]. Medications, including glucocorticoids, aggravate this impairment [4, 5]. Consequently, profound fatigue and exercise intolerance severely reduce health-related quality of life [6].

While pediatric UC quality of life, psychological well-being, and growth are well-documented, objective data on physical fitness and motor proficiency remain scarce. To date, none of the studies in this population has used a comprehensive, standardized battery of tests to assess motor skills. This knowledge gap is clinically significant for three reasons. First, current treatment guidelines for pediatric UC primarily target mucosal healing and symptom control but do not address functional motor recovery—leaving a potential gap in long-term physical health outcomes. Second, unrecognized motor deficits may prevent children from engaging in physical activity, which has been associated with improved quality of life and reduced fatigue in pediatric IBD [7], and may also offer benefits for body composition and disease control. Third, without objective data, clinicians cannot identify which children are at risk for motor impairment and would benefit from targeted rehabilitation. Therefore, identifying whether such deficits exist and which motor domains are affected is a crucial first step toward developing evidence-based rehabilitation strategies.

This study therefore aimed to (i) objectively compare motor proficiency and handgrip strength between children with UC and healthy controls using standardized tests and (ii) analyze the association between disease activity markers and motor outcomes within the UC group. We hypothesized that (i) children with UC would exhibit specific motor proficiency deficits compared to healthy controls, and (ii) these motor impairments would correlate negatively with objective markers of systemic inflammation rather than solely with clinical symptom scores.

## Methods

### Ethics statement

The study was approved by the Antalya Training and Research Hospital local ethics committee (Approval No. 4/7, Date: 27 February, 2025) and was conducted in accordance with the Declaration of Helsinki. Written informed consent was obtained from parents/legal guardians, and assent was obtained from children aged 12 or above.

### Study design and participants

This cross-sectional study recruited participants between March and September 2025. The patient group consisted of children aged 8 to 17 years with confirmed diagnoses of UC for at least 6 months and with sufficient cooperative skills and physical ability to complete the assessments.

An a priori power analysis was performed using G*Power software (version 3.1.9.7), with the Total Motor Composite Score of the Bruininks-Oseretsky Test of Motor Proficiency (BOT-2) defined as the primary outcome. As no previous studies in pediatric UC have used the BOT-2, we conservatively assumed a large effect size (Cohen’s *d* = 0.8) based on literature in other chronic pediatric conditions [8]. With a two-tailed *α* = 0.05 and power = 80%, the required sample size was calculated as 26 participants per group. To account for potential exclusions and to allow for exploratory subgroup analyses, we aimed to recruit approximately 50 participants per group.

Patients were recruited from the Antalya Training and Research Hospital pediatric gastroenterology outpatient clinic. Forty-nine of the 67 eligible patients agreed to participate. The control group consisted of 41 healthy children recruited from the local community through advertisements in schools and primary care centers. Controls were frequency-matched to the UC group for age (± 1 year), sex, and neighborhood socioeconomic status. Exclusion criteria for both groups were the presence of any accompanying acute or chronic disease capable of affecting motor performance (including but not limited to neuromuscular disorders, cardiovascular/respiratory disease, fracture, arthritis), any acute infection within the previous 14 days, or lack of consent. For both groups, exclusion of comorbidities was based on detailed medical history taken by a pediatric gastroenterologist (for UC patients) or a study physician (for controls), supplemented by review of available medical records. No routine laboratory tests, neurological examination, or imaging were performed to rule out subclinical conditions. The case-to-control ratio was not predefined. The numbers in the two groups differ because the control group was recruited to match the demographic characteristics of the patient group, rather than to achieve equal group sizes, and all eligible and consenting controls were included.

### Clinical and disease activity assessment

Disease activity in the children with UC was assessed using the Pediatric Ulcerative Colitis Activity Index (PUCAI) (remission < 10, mild 10–34, moderate 35–64, severe ≥ 65) [9] and the Physician’s Global Assessment (PGA) (inactive, mild, moderate, or severe) [10]. Higher PUCAI and PGA scores indicate more severe disease activity. We defined disease extent per the Paris classification (E1: proctitis, E2: left-sided colitis, E3: extensive colitis, E4: pancolitis) [11].

### Sociodemographic and anthropometric data

A sociodemographic data form collected information concerning the child’s age and sex, parental ages and education levels, marital status, and family monthly income. Income was categorized based on the national gross minimum wage (MW): “poor” (< 1 MW), “average” (1–3 MW), and “good” (> 3 MW). Anthropometric measurements (height and weight) were taken with the children wearing light clothing and barefoot. BMI was calculated as weight (kg)/height^2^ (m^2^). The BMI-for-age z-score (BMI z-score) was calculated using World Health Organization Anthro Plus software (version 1.0.4) for children.

### Biochemical analyses

Venous blood samples were obtained to assess inflammatory and nutritional status. Measured parameters included hemoglobin, hematocrit, C-reactive protein (CRP), albumin, and erythrocyte sedimentation rate (ESR).

### Assessment of motor proficiency

Motor proficiency was evaluated using the complete form BOT-2 [12]. This yields four motor composite scores (Fine Manual Control, Manual Coordination, Body Coordination, and Strength and Agility) and eight subscale scores (Fine Motor Precision, Fine Motor Integration, manual dexterity, upper limb coordination, bilateral coordination, balance, running speed and agility, and strength), providing a comprehensive assessment of both fine and gross motor skills. For all BOT-2 scores, higher values indicate better motor proficiency. The test was administered individually in a quiet, well-lit room in the hospital’s outpatient clinic. Each session lasted approximately 45–60 min, and tasks were presented in a quasi-randomized order as recommended in the manual. Scoring followed the standardized procedures described in the BOT-2 manual, converting raw scores to age-adjusted scale scores and composite scores. The test has demonstrated strong psychometric properties, including high internal consistency and reliability (coefficients > 0.80), as well as good validity for assessing motor proficiency in children with chronic conditions [12]. All assessments were performed by experienced investigators (with over 10 years’ experience) blinded to the children’s clinical status. In order to ensure consistency, the BOT-2 was administered in a single session for each child, the order of tasks following a quasi-randomized sequence. The intra‑ and inter-rater reliability of the investigators for the BOT-2 were excellent, with intraclass correlation coefficients (ICC) ranging from 0.94 to 0.96, calculated based on a subset of 20 randomly selected assessments from the current study sample.

### Assessment of handgrip strength

Handgrip strength was measured using a Jamar hydraulic hand dynamometer. Before measurement, the child was positioned with the elbows flexed to 90° and the forearms in a neutral position. All measurements were performed in the same quiet outpatient clinic setting. The child was then asked to squeeze the dynamometer with the hands at the maximum force for at least 2–3 s, without touching the body. Three trials were performed for each hand with a 1-min rest between trials. The highest value (in Newtons) from the three trials for each hand was recorded for analysis. Values in Newtons were collected for both the dominant and non-dominant hands [13].

### Assessment of physical activity

The Physical Activity Questionnaire for Children or Adolescents (PAQ-C/A), a validated self-report instrument, was used to assess habitual physical activity levels [14]. The questionnaire was completed by the participants in a quiet room under the supervision of the study investigators. The questionnaire has been widely used in previous studies to evaluate the general physical activity levels of children and adolescents [15]. The PAQ-C is typically administered to children aged 8 to 14 and consists of nine items yielding 7-day recall regarding the type and frequency of activities performed during leisure time, physical education classes and recess, immediately after school, in the evenings, and at weekends. The PAQ-A is a modified version of the PAQ-C for adolescents aged over 14, containing the same items, but excluding the question about activities during recess. In both questionnaires, each item is scored from 1 to 5 to obtain item-specific composite activity scores. Higher total scores reflect higher levels of habitual physical activity. The validity and reliability of the Turkish-language version have previously been established [16].

### Statistical analysis

Statistical analyses were performed using IBM SPSS Statistics for Windows version 26.0 software (IBM Corp., Armonk, NY, USA). The normality of distribution of continuous variables was assessed using the Shapiro–Wilk test. Descriptive statistics are presented as mean and standard deviation (SD) for normally distributed variables and as median and interquartile range (IQR) values for non-normally distributed variables. Categorical variables are expressed as numbers and percentages.

The False Discovery Rate (FDR) was controlled in order to account for multiple comparisons across the correlated primary motor outcomes. The Benjamini–Hochberg procedure was applied to the *p* values obtained from the group comparisons of all BOT-2 composite and subscale scores (both unadjusted and adjusted analyses) to control the FDR. Corrected *p* values (denoted as *q*-values) below 0.05 were considered statistically significant for these motor domain comparisons. Handgrip strength and PAQ outcomes were analyzed separately and were not included in the same FDR correction procedure.

(i) Comparisons between UC patients and healthy controls:

Unadjusted (bivariate) comparisons between the UC and healthy control groups were performed using the independent samples *t*-test or the Mann–Whitney *U* test. Categorical variables were compared using the chi-square test or Fisher’s exact test, as appropriate.

(ii) Adjusted comparisons between UC patients and healthy controls:

A series of multivariate analyses were conducted in order to assess the independent association of UC diagnosis with motor proficiency, physical activity, and handgrip strength while controlling for potential sociodemographic and anthropometric confounders. A general linear model (univariate analysis of covariance) was applied for each continuous dependent variable (i.e., each BOT-2 subscale score, the PAQ score, and handgrip strength measures). In these models, the specific group (UC or control) was entered as a fixed factor, with the UC group set as the reference category. Age, sex, parental education level (dichotomized based on whether at least one parent had a university degree or not), family income level (dichotomized as low vs. medium/high), and BMI z-score were included as covariates. Disease-specific parameters such as hemoglobin and inflammatory markers (CRP, ESR) were intentionally excluded from the multivariate models to avoid overadjustment bias, as these variables are considered mediators within the causal pathway of UC rather than independent confounders. The results of these adjusted analyses are presented as unstandardized regression coefficients (*B*) with 95% confidence intervals (CIs), partial eta squared (*η*^2^) as a measure of effect size, and the adjusted coefficient of determination (adjusted *R*^2^). The *p* values for the motor proficiency outcomes from these adjusted models were also corrected for multiple comparisons using the same FDR procedure.

(iii) Analysis of disease activity within the UC patient group:

In the UC patient group, Spearman’s rank correlation coefficient (*ρ*) was used to examine bivariate associations between clinical disease activity indices (PUCAI and PGA), laboratory markers of inflammation (CRP, ESR, albumin, and hemoglobin), and all outcome measures (BOT-2 motor scores, PAQ score, and handgrip strength measures). A two-sided *p* value of < 0.05 was considered statistically significant, except for the motor proficiency outcomes in the group comparisons, for which a FDR-corrected *q*-value < 0.05 was used.

## Results

### Participant characteristics

The study involved 90 children, 49 with UC and 41 controls. The groups’ demographic and clinical characteristics are presented in Table 1. The groups were well matched in terms of age and sex. The median disease duration in the UC group was 23 months, and the majority were in remission or had mild disease activity.

**Table 1:** The demographic, clinical, and laboratory characteristics of the children with ulcerative colitis and healthy controls

	Controls (***n*** = 41)	Children with UC (***n*** = 49)	***p*** value
Age, years	14.6 ± 2.8 (817)	15.3 ± 2.9 (8–17)	0.199
Sex, male, *n* (%)	17 (41.5)	30 (61.2)	0.099
Maternal age	42.6 ± 5.5	44.4 ± 6.7	0.186
Paternal age	46.8 ± 6.2	49.0 ± 6.7	0.121
Maternal education, *n* (%)			
Illiterate	2 (4.9)	3 (6.1)	
Elementary	15 (36.6)	23 (46.9)	0.711
High school	18 (43.9)	16 (32.7)	
Higher education or beyond	6 (14.6)	7 (14.3)	
Paternal education, *n* (%)			
Illiterate	1 (2.5)	0 (0)	
Elementary	14 (35.0)	18 (36.7)	0.691
High school	18 (45.0)	24 (49.0)	
Higher education or beyond	7 (17.5)	7 (14.3)	
Marital status, *n* (%)			
Married	39 (95.1)	45 (91.8)	0.685
Divorced	2 (4.9)	4 (8.2)	
Household income, *n* (%)			
Good	11 (26.8)	10 (20.4)	
Average	23 (56.1)	22 (44.9)	0.169
Poor	7 (17.1)	17 (34.7)	
BMI, kg/m^2^	19.7 ± 3.9	19.7 ± 4.2	0.995
BMI z‐score	−0.24 (−1.36 to 0.52)	−0.21 (−1.03 to 0.61)	0.756
Laboratory data			
Hemoglobin, g/L	13.1 ± 1.4	12.4 ± 2.5	< 0.001
CRP, mg/L	0.4 (0.3–2.2)	1.5 (0.5–4.1)	0.036
Albumin, g/L	46.5 ± 3.3	44.0 ± 4.8	0.015
ESR, mm/h	21.0 (13.0–25.0)	20.0 (13.5–28.0)	0.254
Duration of disease, months		23 (13–38)	
Biological agent use, *n* (%)			
Yes		19 (38.8)	
No		30 (61.2)	
Disease characteristics, *n* (%)			
E1 ulcerative proctitis		2 (4.1)	
E2 left‐sided colitis		9 (18.4)	
E3 extensive colitis		10 (20.4)	
E4 pancolitis		28 (57.1)	
Disease activity			
PUCAI, *n* (%)			
Remission		28 (57.1)	
Mild activity		14 (28.6)	
Moderate activity		2 (4.1)	
Severe disease		5 (10.2)	
PGA, *n* (%)			
Inactive disease		20 (40.8)	
Mild disease		17 (34.7)	
Moderate disease		5 (10.2)	
Severe disease		7 (14.3)	

Group comparisons were performed using the independent samples *t*-test, Mann–Whitney *U* test, chi-square test, or Fisher’s exact test, as appropriate. Statistically significant *p* values are highlighted in bold. Continuous variables are expressed as mean (standard deviation) or median (interquartile range) values and categorical variables as number (percentage)

*UC*, ulcerative colitis; *BMI*, body mass index; *CRP*, C‐reactive protein; *ESR*, erythrocyte sedimentation rate; *PUCAI*, Pediatric Ulcerative Colitis Activity Index; *PGA*, Physician Global Assessment; *IQR*, interquartile range; *SD*, standard deviation

### Unadjusted motor proficiency and strength comparisons

Unadjusted comparisons (Table 2) revealed that children with UC registered significantly lower scores across most motor proficiency domains. After applying FDR correction, significant deficits (*q* < 0.05) were observed in Manual Coordination, Manual Dexterity, Upper Limb Coordination, Body Coordination, Bilateral Coordination, Balance, Strength and Agility, Running Speed and Agility, and Strength, culminating in a significantly lower Total Motor Composite Score. In contrast, no significant differences were observed in the fine motor domains of Fine Manual Control, Fine Motor Precision, or Fine Motor Integration. The UC patients demonstrated lower bilateral handgrip strength values compared to the controls (*p* < 0.05 for both).

**Table 2:** Motor proficiency, physical activity, and handgrip strength assessment in the ulcerative colitis and control groups

Variable	Control group (***n*** = 41)	UC patients (***n*** = 49)	***p*** value	***q***-value (FDR adjusted)
BOT-2				
Fine Manual Control	18.1 ± 5.1	16.9 ± 5.1	0.201	0.218
Fine Motor Precision	10.0 (7.0–12.0)	8.0 (7.0–11.0)	0.136	0.161
Fine Motor Integration	7.0 (6.0–8.0)	7.0 (6.0–8.5)	0.888	0.888
Manual Coordination	32.1 ± 6.4	25.1 ± 7.4	< 0.001	0.013
Manual Dexterity	17.0 (14.0–19.0)	12.0 (9.0–15.0)	< 0.001	0.006
Upper Limb Coordination	15.0 (11.0–20.5)	12.0 (9.5–17.0)	0.005	0.007
Body Coordination	29.9 ± 6.2	22.5 ± 6.1	< 0.001	0.004
Bilateral Coordination	17.0 (12.0–18.0)	8.0 (6.0–11.0)	< 0.001	0.003
Balance	15.0 (12.0–19.0)	13.0 (9.5–16.5)	0.031	0.004
Strength and Agility	30.0 ± 6.9	23.9 ± 6.4	< 0.001	0.003
Running Speed and Agility	14.0 (11.0–16.0)	11.0 (8.0–14.0)	0.001	0.002
Strength	17.0 (13.0–19.5)	12.0 (10.0–16.0)	0.001	0.002
Total Motor Composite Score	45.1 ± 7.0	37.4 ± 5.4	< 0.001	0.002
PAQ				
PAQ total score	1.56 (1.12–2.26)	1.37 (1.15–2.01)	0.554	-
Handgrip strength				
Dominant hand, *N*	281.1 (178.1–339.9)	195.6 (144.7–245.1)	0.017	-
Non-dominant hand, *N*	254.9 (161.8–328.5)	176.3 (123.0–217.4)	0.011	-

Data are presented as mean (standard deviation) for normally distributed continuous variables or median (interquartile range) for non-normally distributed continuous variables, based on the Shapiro–Wilk test. Group comparisons were performed using the independent samples *t*-test for normally distributed variables and the Mann–Whitney *U* test for non-normally distributed variables. For the multiple BOT-2 score comparisons, the FDR was controlled using the Benjamini–Hochberg procedure. Corrected *q*-values < 0.05 were considered statistically significant and are highlighted in bold. The PAQ and handgrip strength measures were analyzed separately and are not included in the FDR correction (indicated by “- “). Uncorrected *p* values are also shown for completeness

*UC*, ulcerative colitis; *BOT-2*, Bruininks-Oseretsky Test of Motor Proficiency-2; *PAQ*, Physical Activity Questionnaire; *N*, Newtons (unit of measure); *IQR*, interquartile range; *SD*, standard deviation; *FDR*, False Discovery Rate

### Adjusted comparisons between groups: multivariate analysis

A series of general linear model analyses were performed in order to control for potential confounding sociodemographic and anthropometric factors. After adjustment for age, sex, parental education, family income, and BMI z-score, the diagnosis of UC remained independently associated with lower scores in specific motor domains (Table 3). FDR correction confirmed deficits with large effect sizes in Manual Coordination (adjusted *B* =  − 6.72, *q* = 0.013), Body Coordination (adjusted *B* =  − 6.84, *q* = 0.004), and Bilateral Coordination (adjusted *B* =  − 5.65, *q* = 0.003). Significant deficits were also observed in Manual Dexterity (*q* = 0.006), Upper Limb Coordination (*q* = 0.013), Strength and Agility (*q* = 0.003), Running Speed and Agility (*q* = 0.002), Strength (*q* = 0.005), and the Total Motor Composite Score (*q* = 0.002). Conversely, Fine Manual Control and Balance lost significance (*q* > 0.05). The adjusted analysis for handgrip strength showed no significant difference for dominant (*B* =  − 16.59, *p* = 0.278) or non-dominant hands (*B* =  − 26.05, *p* = 0.091). Adjusted models revealed no independent association between UC diagnosis and PAQ score (*B* =  − 0.02, *p* = 0.909) or physical activity status (OR = 1.96, *p* = 0.234).

**Table 3:** Adjusted comparisons of motor proficiency, physical activity, and handgrip strength between the children with ulcerative colitis and healthy controls

Variable	Adjusted ***B*** (95% CI)	***q***-value (FDR adjusted)	Partial ***η***^2^	Adjusted ***R***^2^
BOT-2				
Fine Manual Control	− 0.42 (− 2.46, 1.62)	0.805	0.002	0.183
Fine Motor Precision	− 0.27 (− 1.96, 1.42)	0.813	0.001	0.161
Fine Motor Integration	− 0.15 (− 1.15, 0.85)	0.763	0.001	0.118
Manual Coordination	− 6.72 (− 9.83, − 3.60)	0.013	0.181	0.191
Manual Dexterity	− 4.20 (− 6.13, − 2.26)	0.006	0.183	0.188
Upper Limb Coordination	− 2.64 (− 4.60, − 0.68)	0.013	0.079	0.063
Body Coordination	− 6.84 (− 9.60, − 4.09)	0.004	0.227	0.255
Bilateral Coordination	− 5.65 (− 7.57, − 3.72)	0.003	0.291	0.302
Balance	− 1.47 (− 3.32, 0.37)	0.151	0.030	0.067
Strength and Agility	− 5.74 (− 8.66, − 2.82)	0.003	0.155	0.186
Running Speed and Agility	− 2.71 (− 4.33, − 1.08)	0.002	0.117	0.089
Strength	− 2.77 (− 4.57, − 0.97)	0.005	0.102	0.237
Total Motor Composite Score	− 6.64 (− 9.21, − 4.06)	0.002	0.240	0.360
PAQ				
PAQ total score	−0.02 (− 0.32, 0.28)	0.909 (p)	0.000	0.034
Handgrip strength				
Dominant hand, *N*	− 16.59 (− 46.84, 13.65)	0.278 (p)	0.015	0.165
Non-dominant hand, *N*	− 26.05 (− 56.38, 4.27)	0.091 (p)	0.037	0.163

All models were adjusted for age, sex, parental education (at least one university graduate), family income level (low vs. medium/high), and BMI z-score. Negative *B* values indicate lower scores in the UC group compared to the control group. For the BOT-2 scores, the FDR was controlled using the Benjamini–Hochberg procedure. Corrected *q*-values < 0.05 were considered statistically significant and are highlighted in bold. For PAQ and handgrip strength outcomes (marked with “(p)”), which were analyzed separately, the table shows uncorrected *p* values. Partial *η*^2^ effect size: small ≥ 0.01, medium ≥ 0.06, large ≥ 0.14

BMI z‑score was included as a covariate to test the independence of the association between UC and motor outcomes from anthropometric status. However, because BMI may lie on the causal pathway between UC and muscle function (i.e., a mediator rather than a pure confounder), the adjusted analyses represent a conservative estimate; unadjusted comparisons (Table 2) may better reflect the total disease burden

*UC*, ulcerative colitis; *BOT-2*, Bruininks-Oseretsky Test of Motor Proficiency-2; *PAQ*, Physical Activity Questionnaire; *N*, Newtons (unit of measure); *B*, unstandardized regression coefficient; *CI*, confidence interval; *FDR*, False Discovery Rate

### Disease activity and motor outcomes within the UC group

Within the UC patient group, Spearman’s analyses revealed significant associations between disease activity and motor outcomes (Table 4). Systemic inflammation markers correlated negatively: higher ESR with lower non-dominant handgrip (*ρ* =  − 0.533, *p* < 0.001), dominant handgrip strength (*ρ* =  − 0.414, *p* = 0.006), Strength and Agility (*ρ* =  − 0.414, *p* = 0.003), and Running Speed and Agility (*ρ* =  − 0.422, *p* = 0.003). Higher CRP correlated with lower Strength and Agility (*ρ* =  − 0.400, *p* = 0.004) and Strength (*ρ* =  − 0.382, *p* = 0.007). Lower albumin was associated with poorer Fine Manual Control (*ρ* = 0.399, *p* = 0.004), Fine Motor Integration (*ρ* = 0.396, *p* = 0.005), and Running Speed and Agility (*ρ* = 0.346, *p* = 0.016). Additionally, higher hemoglobin levels showed a significant positive correlation with greater handgrip strength for both the non-dominant (*ρ* = 0.427, *p* = 0.005) and dominant (*ρ* = 0.352, *p* = 0.019) hands. The complete correlation matrix, including all non-significant correlations, is presented in Supplementary Table 1. To evaluate the potential impact of treatment on motor outcomes, we compared children with UC who were receiving biological agents (*n* = 19) to those who were not (*n* = 30). No significant differences were observed in any of the BOT-2 composite or subscale scores, handgrip strength measures, or PAQ scores (all *p* > 0.05). Disease duration showed significant positive correlations with several motor outcomes in unadjusted analyses, including the Total Motor Composite Score (*ρ* = 0.349, *p* = 0.014), Strength and Agility (*ρ* = 0.441, *p* = 0.002), Bilateral Coordination (*ρ* = 0.339, *p* = 0.017), and Manual Dexterity (*ρ* = 0.330, *p* = 0.021). However, these associations were largely driven by age, as children with longer disease duration were older; after controlling for age in the multivariate models (Table 3), no independent effect of disease duration remained.

**Table 4:** Significant correlations between disease activity parameters and motor outcomes in the children with ulcerative colitis

Disease activity parameter	Motor or strength outcome	Spearman’s *ρ*	*p* value
PGA	Balance	− 0.315	0.028
CRP, mg/L	Strength and Agility	− 0.400	0.004
	Strength	− 0.382	0.007
ESR, mm/h	Strength and Agility	− 0.414	0.003
	Running Speed and Agility	− 0.422	0.003
	Non-Dominant Handgrip Strength	− 0.533	< 0.001
	Dominant Handgrip Strength	− 0.414	0.006
Albumin, g/L	Fine Manual Control	0.399	0.004
	Fine Motor Integration	0.396	0.005
	Running Speed and Agility	0.346	0.016
Hemoglobin, g/L	Non-Dominant Handgrip Strength	0.427	0.005
	Dominant Handgrip Strength	0.352	0.019

This table presents all statistically significant bivariate associations (*p* < 0.05) between disease activity parameters and motor outcomes within the ulcerative colitis patient group (*n* = 49), as assessed by Spearman’s rank correlation coefficient (*ρ*). PUCAI and disease extent (Paris classification) exhibited no significant correlations with any motor outcome (all *p* > 0.05) and are therefore not listed

*PUCAI*, Pediatric Ulcerative Colitis Activity Index; *PGA*, Physician Global Assessment; *CRP*, C-reactive Protein; *ESR*, erythrocyte sedimentation rate

## Discussion

The study findings showed that children with UC have significantly lower motor proficiency compared to healthy controls. This finding supports the growing body of literature indicating impaired motor function in pediatric chronic diseases. Kantanen et al. [17] reported that children with IBD exhibit low cardiorespiratory and neuromuscular fitness, with particular deficits in tests such as handgrip strength, the standing long jump, and the shuttle run. Similarly, reduced motor proficiency compared to healthy controls has been reported in children with acute lymphoblastic leukemia and intestinal failure [8, 18]. These results indicate that, similarly to other chronic diseases, UC can adversely affect motor development.

The most striking finding of this study is the distinct dissociation within the UC motor profile: gross motor skills and coordination-dependent tasks are severely affected, whereas fine motor skills remain largely preserved. Multivariate analyses confirmed that UC diagnosis had no significant negative effect on fine motor areas after controlling for age, sex, parental education, and BMI z-score. The low PAQ scores in both groups indicate a general trend to a sedentary lifestyle. In this context, children with UC may spend as much time as their healthy peers engaged in sedentary activities requiring fine motor skills, such as schoolwork and computer use. This frequent engagement may promote the preservation of the related neural pathways [19]. This preservation suggests the disease does not cause dyspraxia or general neurological degradation.

In contrast to fine motor skills, we detected marked impairment in gross motor functions in children with UC, particularly in bilateral coordination and balance. The decline in bilateral coordination suggests a disruption in the ability to sequence and execute complex motor tasks. The loss of balance may point to an issue with the central integration of vestibular input, visual cues, and proprioception. Multiple mechanisms may underlie this impairment. First, the “protective” flexion posture adopted due to abdominal pain and cramps may, over time, lead to trunk muscle weakness and postural instability [6, 17]. Second, although not directly measured in the present study, micronutrient deficiencies common in UC (such as vitamin B12), which can affect nerve conduction, may be a contributing factor [20, 21].

The decline in the Manual Coordination domain may initially appear contradictory to the preserved fine motor skills. However, in the BOT-2 test, this subdomain assesses not only manual dexterity but also object control, reaction time, and speed [22]. This finding indicates that the core problem in children with UC is not movement execution itself, but the ability to perform it forcefully and in a timely manner. This suggests inflammation-induced “psychomotor slowing” or reduced fast-twitch muscle fiber capacity [23, 24]. The decline in the Strength and Agility domain further corroborates this physical reserve limitation. This area tests anaerobic power and endurance through items such running speed and standing long jump. These impairments represent significant factors that directly threaten children’s participation in sports, their capacity to play with peers, and, consequently, their overall quality of life.

Absolute handgrip strength was significantly lower in UC patients, consistent with “muscle-bone unit” deficits in chronic inflammatory diseases [25]. However, the attenuation of these differences after adjusting for BMI z‑score should be interpreted cautiously. Because BMI is reduced in children with UC as a consequence of chronic inflammation and malnutrition, adjusting for it may remove part of the disease’s true effect on muscle strength (overadjustment bias). Thus, the unadjusted comparisons (Table 2), which showed significantly lower handgrip strength in the UC group, likely better reflect the clinically relevant deficit. Our finding underscores that disease‑induced growth impairment contributes to reduced strength but does not rule out additional neuromuscular mechanisms, such as inflammation‑driven muscle catabolism or reduced fast‑twitch fiber recruitment.

One of the most important and clinically significant findings of this study is the strong negative association determined between the objective systemic inflammation marker, ESR, and motor performance. ESR emerged as the strongest independent negative predictor of muscle strength and agility. Unlike CRP, ESR reflects cumulative inflammatory burden [26]; thus, chronically mild elevations may indicate “silent” sarcopenia driven by cytokines (e.g., interleukin-6), which increase muscle protein breakdown and suppress its synthesis [27]. Another key finding is the positive correlation between hemoglobin levels and handgrip strength. Anemia in children with UC is frequently a combination of iron deficiency and anemia of chronic disease [28]. This association is biologically plausible, as reduced hemoglobin may impair oxygen delivery to tissues, thereby potentially affecting muscle endurance and strength. However, the cross‑sectional design of our study precludes causal inference, and the observed relationship should be interpreted as an association rather than a direct effect.

Despite the majority of our patients being in clinical remission according to the PUCAI, we observed significant motor deficits. However, because of the cross‑sectional design, we cannot determine whether these deficits predate diagnosis, emerge during active disease, or persist despite clinical remission. The absence of a correlation between PUCAI and motor outcomes, together with the significant correlations observed with ESR, is clinically informative. It is well established that many patients with UC achieve clinical remission despite persistent endoscopic inflammation (so‑called “discordant remission”) [9]. Our findings suggest that this subclinical inflammation—reflected by elevated ESR—may contribute to ongoing motor impairment. Therefore, objective inflammatory markers such as ESR may be more informative than symptom scores for identifying children at risk for motor deficits. Prospective longitudinal studies following children from diagnosis are needed to establish temporal relationships and to determine whether targeted rehabilitation can reverse these deficits.

Another significant finding of this study is the absence of a significant difference in self-reported activity levels, as measured by the PAQ, between children with UC and healthy controls, with scores being low in both groups. Two primary explanations are plausible. First, the generalized childhood sedentariness caused by modern lifestyles may statistically mask disease-specific inactivity in UC. Second, and more critically, there is the conceptual difference between the assessment tools used. The PAQ inquires into the frequency of activity but does not directly assess its intensity, duration, or quality. In contrast, the BOT-2 directly and objectively measures the quality of motor proficiency and performance. A child with UC may report “participating” in a physical education class on the questionnaire and score points accordingly, yet may do so with only limited effort. This finding underscores the need for objective performance tests and, where possible, activity monitoring instead of self-reports in the assessment of physical activity.

These findings highlight a gap in pediatric UC management. While available medical treatments can achieve remission, this does not guarantee the automatic recovery of muscle function and motor skills. The fact that the great majority of our patients were inactive and that their motor skills lagged behind their peers indicates that these children are at a severe risk of long-term musculoskeletal health. Previous research suggests that regular exercise may produce anti-inflammatory effects in IBD and increase bone density [29]. However, the balance, coordination, and strength deficits in children with UC may prevent them from participating effectively in standard sports programs. It is therefore essential to move beyond generic “move more” advice. Multidisciplinary teams must design targeted, progressive rehabilitation programs addressing specific deficits like balance and agility. This study represents a step towards building the necessary evidence base for developing such programs.

This study has several strengths. To our knowledge, it is the first to comprehensively assess motor proficiency in children with UC using the complete form of the BOT‑2, a well‑validated and standardized tool that evaluates both fine and gross motor domains. The inclusion of a closely matched healthy control group and the adjustment for important confounders (age, sex, socioeconomic status, BMI z‑score) strengthen the validity of our findings. Furthermore, the concurrent assessment of disease activity, laboratory markers, and objective physical performance measures allows a multidimensional evaluation of motor impairment in this population. Several limitations should be acknowledged. First, the cross‑sectional design precludes causal inferences; we cannot determine whether motor deficits predate diagnosis, emerge during active disease, or persist despite remission. Second, we did not assess mucosal healing via endoscopy or fecal calprotectin; because clinical remission (PUCAI) can dissociate from endoscopic activity, future studies should incorporate objective markers of intestinal inflammation. Third, exclusion of comorbidities (e.g., neuromuscular disorders) relied on medical history and parent/child interview rather than systematic neurological examination or laboratory screening, which may have introduced bias. Fourth, we lacked sufficient power for subgroup analyses by medication class (e.g., biologics vs. conventional therapy) and did not collect detailed data on cumulative corticosteroid exposure; only two patients had a history of surgery, precluding meaningful analysis of these treatment‑related factors. Fifth, physical activity was assessed via self‑report (PAQ); objective methods such as accelerometry are needed. Sixth, we did not perform detailed body composition analysis (e.g., fat‑free mass by DXA or bioimpedance) nor measure specific micronutrient levels (e.g., vitamin D, B12, iron), which may influence motor performance.

Future longitudinal studies are warranted to establish causal relationships, to investigate the impact of cumulative corticosteroid exposure and surgical history on motor outcomes, and to evaluate whether targeted rehabilitation programs can effectively improve motor function in this population. Additionally, studies incorporating objective physical activity monitoring (e.g., accelerometry) and detailed body composition analysis would provide deeper insight into the mechanisms underlying motor impairment in children with UC.

## Conclusion

In conclusion, this study shows that children with pediatric UC experience clinically significant deficits in fundamental motor functions, including handgrip strength, body coordination, balance, and agility, even when the disease is in clinical remission. The preservation of fine motor skills points to a selective mechanism primarily targeting the gross motor system and muscle metabolism. Consequently, pediatric UC management must extend beyond clinical remission to include functional recovery. Integrating early motor screening and individualized physical rehabilitation into routine care is essential to reduce long-term physical disability risks and optimize quality of life.

## Supplementary Information

Below is the link to the electronic supplementary material.

ESM 1 (DOCX 18.3 KB)

## Data Availability

The datasets used and/or analyzed in the current study are available from the corresponding author upon reasonable request.

## References

[1] Weidner J, Kern I, Reinecke I et al (2024) A systematic review and meta‐regression on international trends in the incidence of ulcerative colitis in children and adolescents associated with socioeconomic and geographic factors. Eur J Pediatr 183(4):1723–1732. 10.1007/s00431-024-05428-3 38231235 10.1007/s00431-024-05428-3 PMC11001685

[2] Long D, Wang C, Huang Y, Mao C, Xu Y, Zhu Y (2024) Changing epidemiology of inflammatory bowel disease in children and adolescents. Int J Colorectal Dis 39(1):73. 10.1007/s00384-024-04640-9 38760622 10.1007/s00384-024-04640-9 PMC11101569

[3] Wine E, Aloi M, Van Biervliet S et al (2025) Management of paediatric ulcerative colitis, Part 1: Ambulatory care—an updated evidence-based consensus guideline from the European Society of Paediatric Gastroenterology, Hepatology and Nutrition and the European Crohn’s and Colitis Organisation. J Pediatr Gastroenterol Nutr 81(3):765–815. 10.1002/jpn3.70097 40677018 10.1002/jpn3.70097 PMC12408984

[4] Rudra S, Kennedy K, Neukrug S et al (2025) Prevalence and predictors of low bone mineral density in pediatric inflammatory bowel disease. J Pediatr Gastroenterol Nutr 81(1):34–42. 10.1002/jpn3.70047 40296588 10.1002/jpn3.70047 PMC12665282

[5] Bouhuys M, Lexmond WS, van Rheenen PF (2023) Pediatric inflammatory bowel disease. Pediatrics 151(1):e2022058037. 10.1542/peds.2022-058037 36545774 10.1542/peds.2022-058037

[6] Bevers N, Van de Vijver E, Hanssen A et al (2023) Fatigue and physical activity patterns in children with inflammatory bowel disease. J Pediatr Gastroenterol Nutr 77(5):628–633. 10.1097/MPG.0000000000003905 37494540 10.1097/MPG.0000000000003905

[7] Trivić I, Sila S, Mišak Z et al (2023) Impact of an exercise program in children with inflammatory bowel disease in remission. Pediatr Res 93(7):1999–2004. 10.1038/s41390-022-02362-8 36319697 10.1038/s41390-022-02362-8 PMC9628325

[8] Yildiz Kabak V, Ekinci Y, Atasavun Uysal S, Cetin M, Duger T (2021) Motor and basic cognitive functions in children with acute lymphoblastic leukemia undergoing induction or consolidation chemotherapy. Percept Mot Skills 128(3):1091–1106. 10.1177/00315125211002065 33730934 10.1177/00315125211002065

[9] Turner D, Otley AR, Mack D et al (2007) Development, validation, and evaluation of a pediatric ulcerative colitis activity index: a prospective multicenter study. Gastroenterology 133:423–432. 10.1053/j.gastro.2007.05.029 17681163 10.1053/j.gastro.2007.05.029

[10] Physician Global Assessment: ImproveCareNow. Available online: https://d3n8a8pro7vhmx.cloudfront.net/improvecarenow/pages/283/attachments/original/1456525231/PGA_Clinical_Guidelines.pdf?1456525231.

[11] Levine A, Griffiths A, Markowitz J et al (2011) Pediatric modification of the Montreal classification for inflammatory bowel disease: the Paris classification. Inflamm Bowel Dis 17:1314–21. 10.1002/ibd.21493 21560194 10.1002/ibd.21493

[12] Bruininks RH, Bruininks BD (2005) BOT2: Bruininks-Oseretsky test of motor proficiency. Pearson, Assessments

[13] Guesta-Vargas A, Hilgenkamp T (2015) Reference values of grip strength measured with a Jamar Dynamometer in 1526 adults with intellectual disabilities and compared to adults without intellectual disability. PLoS One 10(6):e0129585. 10.1371/journal.pone.0129585 26053852 10.1371/journal.pone.0129585 PMC4460022

[14] Kowalski KC, Crocker PR, Donen RM (2004) The physical activity questionnaire for older children (PAQ-C) and adolescents (PAQ-A) manual. College of Kinesiology, University of Saskatchewan 87(1):1–38

[15] Kawalec A, Mozrzymas R, Domżol A et al (2024) Physical activity and its potential determinants in obese children and adolescents under specialist outpatient care-a pilot cross-sectional study. Healthcare Basel 12(2):260. 10.3390/healthcare12020260 38275539 10.3390/healthcare12020260 PMC10815763

[16] Erdim L, Ergün A, Kuğuoğlu S (2019) Reliability and validity of the Turkish version of the Physical Activity Questionnaire for Older Children (PAQ-C). Turk J Med Sci 49:162–169 30764593 10.3906/sag-1806-212 PMC7371234

[17] Kantanen S, Räsänen K, Kolho KL et al (2025) Impaired physical fitness in pediatric inflammatory bowel disease. J Pediatr Gastroenterol Nutr 81(2):275–285. 10.1002/jpn3.70091 40444501 10.1002/jpn3.70091 PMC12314574

[18] So S, Patterson C, Evans C, Wales PW (2019) Motor proficiency and generalized self-efficacy toward physical activity in children with intestinal failure. J Pediatr Gastroenterol Nutr 68(1):7–12. 10.1097/MPG.0000000000002107 30052565 10.1097/MPG.0000000000002107

[19] Granados-Delgado P, Casares-López M, Martino F, Anera RG, Castro-Torres JJ (2024) The role of visual performance in fine motor skills. Life Basel 14(11):1354. 10.3390/life14111354 39598153 10.3390/life14111354 PMC11595507

[20] Nishikawa H, Nakamura S, Miyazaki T et al (2021) Inflammatory bowel disease and sarcopenia: its mechanism and clinical importance. J Clin Med 10(18):4214. 10.3390/jcm10184214 34575326 10.3390/jcm10184214 PMC8470813

[21] Gold SL, Manning L, Kohler D, Ungaro R, Sands B, Raman M (2023) Micronutrients and their role in inflammatory bowel disease: function, assessment, supplementation, and impact on clinical outcomes including muscle health. Inflamm Bowel Dis 29(3):487–501. 10.1093/ibd/izac223 36287025 10.1093/ibd/izac223

[22] Tadin Hadjina I, Zivkovic PM, Matetic A et al (2019) Impaired neurocognitive and psychomotor performance in patients with inflammatory bowel disease. Sci Rep 9(1):13740. 10.1038/s41598-019-50192-2 31551482 10.1038/s41598-019-50192-2 PMC6760518

[23] Ergenc I, Ismail Basa C, Uzum A et al (2024) High prevalence of muscle weakness and probable sarcopenia in patients with inflammatory bowel disease. Nutr Clin Pract 39(3):557–567. 10.1002/ncp.11125 38321633 10.1002/ncp.11125

[24] Massironi S, Viganò C, Palermo A et al (2023) Inflammation and malnutrition in inflammatory bowel disease. Lancet Gastroenterol Hepatol 8(6):579–90. 10.1016/S2468-1253(23)00011-0 36933563 10.1016/S2468-1253(23)00011-0

[25] Aljilani B, Tsintzas K, Jacques M, Radford S, Moran GW (2023) Systematic review: sarcopenia in paediatric inflammatory bowel disease. Clin Nutr ESPEN 57:647–654. 10.1016/j.clnesp.2023.08.009 37739718 10.1016/j.clnesp.2023.08.009

[26] Singh G (2024) C-reactive protein and erythrocyte sedimentation rate in inflammatory lesions: Erythrocyte sedimentation rate test is essential in a subset of patients. Am J Clin Pathol 161(3):311. 10.1093/ajcp/aqad125 37842812 10.1093/ajcp/aqad125

[27] Nardone OM, de Sire R, Petito V et al (2021) Inflammatory bowel diseases and sarcopenia: the role of inflammation and gut microbiota in the development of muscle failure. Front Immunol 12:694217. 10.3389/fimmu.2021.694217 34326845 10.3389/fimmu.2021.694217 PMC8313891

[28] Broekaert IJ, Assa A, Borrelli O et al (2025) Approach to anaemia in gastrointestinal disease: a position paper by the ESPGHAN Gastroenterology Committee. J Pediatr Gastroenterol Nutr 80(3):510–532. 10.1002/jpn3.12454 39783775 10.1002/jpn3.12454 PMC11874238

[29] Scheffers LE, Vos IK, Utens EMWJ, Rotterdam Exercise Team (2023) Physical training and healthy diet improved bowel symptoms, quality of life, and fatigue in children with inflammatory bowel disease. J Pediatr Gastroenterol Nutr 77(2):214–221. 10.1097/MPG.0000000000003816 37134004 10.1097/MPG.0000000000003816 PMC10348627

