# Supplementary material for: The evaluation of motor deficits in children with ulcerative colitis: a cross-sectional study
Source: Eur J Pediatr. 2026 May 4;185(5):341. doi: 10.1007/s00431-026-07014-1 (PMC13139249; doi:10.1007/s00431-026-07014-1)
Supplement: Supplementary file 1 — (DOCX 18.3 KB) [file 431_2026_7014_MOESM1_ESM.docx]

**Supplementary Table 1:** Complete correlation matrix between disease activity parameters and motor outcomes in children with ulcerative colitis (n = 49).

| **Parameter** | **Outcome** | **Spearman’s ρ** | **p‑value** |
| --- | --- | --- | --- |
| **CRP** | Strength and Agility | -0.400 | 0.004 |
|  | Strength | -0.382 | 0.007 |
|  | Running Speed and Agility | -0.302 | 0.051 |
|  | Total Motor Composite | -0.223 | 0.055 |
|  | Physical Activity | -0.241 | 0.066 |
|  | Fine Manual Control | 0.038 | 0.795 |
|  | Fine Motor Precision | 0.001 | 0.994 |
|  | Fine Motor Integration | 0.101 | 0.494 |
|  | Manual Coordination | -0.212 | 0.148 |
|  | Manual Dexterity | -0.108 | 0.465 |
|  | Upper Limb Coordination | -0.199 | 0.175 |
|  | Body Coordination | -0.080 | 0.591 |
|  | Bilateral Coordination | -0.181 | 0.219 |
|  | Balance | -0.019 | 0.901 |
|  | Non-Dominant Handgrip Strength | -0.091 | 0.171 |
|  | Dominant Handgrip Strength | -0.218 | 0.561 |
| **Hemoglobin** | Running Speed and Agility | 0.314 | 0.075 |
|  | Strength and Agility | 0.235 | 0.108 |
|  | Total Motor Composite | 0.175 | 0.235 |
|  | Physical Activity | 0.107 | 0.468 |
|  | Fine Manual Control | -0.109 | 0.459 |
|  | Fine Motor Precision | -0.057 | 0.700 |
|  | Fine Motor Integration | -0.159 | 0.279 |
|  | Manual Coordination | 0.073 | 0.623 |
|  | Manual Dexterity | -0.032 | 0.830 |
|  | Upper Limb Coordination | 0.246 | 0.091 |
|  | Body Coordination | 0.174 | 0.236 |
|  | Bilateral Coordination | 0.045 | 0.762 |
|  | Balance | 0.122 | 0.410 |
|  | Strength | 0.114 | 0.439 |
|  | Non-Dominant Handgrip Strength | 0.427 | 0.005 |
|  | Dominant Handgrip Strength | 0.352 | 0.019 |
| **ESR** | Strength and Agility | -0.414 | 0.003 |
|  | Running Speed and Agility | -0.422 | 0.003 |
|  | Strength | -0.257 | 0.054 |
|  | Total Motor Composite | -0.191 | 0.193 |
|  | Physical Activity | -0.155 | 0.294 |
|  | Fine Manual Control | 0.108 | 0.465 |
|  | Fine Motor Precision | 0.056 | 0.708 |
|  | Fine Motor Integration | 0.172 | 0.243 |
|  | Manual Coordination | 0.039 | 0.792 |
|  | Manual Dexterity | 0.073 | 0.623 |
|  | Upper Limb Coordination | -0.131 | 0.376 |
|  | Body Coordination | -0.182 | 0.215 |
|  | Bilateral Coordination | -0.073 | 0.623 |
|  | Balance | -0.214 | 0.144 |
|  | Non-Dominant Handgrip Strength | -0.533 | <0.001 |
|  | Dominant Handgrip Strength | -0.414 | 0.006 |
| **PUCAI** | Balance | -0.196 | 0.051 |
|  | Physical Activity | -0.178 | 0.098 |
|  | Strength and Agility | -0.201 | 0.068 |
|  | Strength | -0.251 | 0.086 |
|  | Running Speed and Agility | -0.241 | 0.099 |
|  | Total Motor Composite | -0.162 | 0.272 |
|  | Fine Manual Control | 0.068 | 0.646 |
|  | Fine Motor Precision | 0.031 | 0.836 |
|  | Fine Motor Integration | 0.116 | 0.434 |
|  | Manual Coordination | -0.041 | 0.782 |
|  | Manual Dexterity | 0.128 | 0.388 |
|  | Upper Limb Coordination | -0.135 | 0.362 |
|  | Body Coordination | -0.126 | 0.394 |
|  | Bilateral Coordination | 0.077 | 0.605 |
|  | Non-Dominant Handgrip Strength | -0.018 | 0.913 |
|  | Dominant Handgrip Strength | -0.082 | 0.602 |
| **PGA** | Balance | -0.315 | 0.028 |
|  | Physical Activity | -0.259 | 0.063 |
|  | Strength and Agility | -0.280 | 0.054 |
|  | Running Speed and Agility | -0.283 | 0.051 |
|  | Total Motor Composite | -0.147 | 0.320 |
|  | Fine Manual Control | 0.092 | 0.534 |
|  | Fine Motor Precision | 0.028 | 0.849 |
|  | Fine Motor Integration | 0.121 | 0.412 |
|  | Manual Coordination | -0.008 | 0.959 |
|  | Manual Dexterity | 0.161 | 0.275 |
|  | Upper Limb Coordination | -0.145 | 0.326 |
|  | Body Coordination | -0.186 | 0.205 |
|  | Bilateral Coordination | 0.044 | 0.766 |
|  | Strength | -0.179 | 0.224 |
|  | Non-Dominant Handgrip Strength | -0.219 | 0.168 |
|  | Dominant Handgrip Strength | -0.128 | 0.413 |
| **Albumin** | Fine Manual Control | 0.399 | 0.004 |
|  | Fine Motor Precision | 0.284 | 0.056 |
|  | Fine Motor Integration | 0.396 | 0.005 |
|  | Physical Activity | 0.248 | 0.061 |
|  | Running Speed and Agility | 0.346 | 0.016 |
|  | Strength and Agility | 0.177 | 0.229 |
|  | Total Motor Composite | -0.095 | 0.519 |
|  | Manual Coordination | -0.116 | 0.432 |
|  | Manual Dexterity | -0.070 | 0.638 |
|  | Upper Limb Coordination | 0.020 | 0.895 |
|  | Body Coordination | -0.103 | 0.486 |
|  | Bilateral Coordination | -0.234 | 0.109 |
|  | Balance | 0.101 | 0.495 |
|  | Strength | 0.080 | 0.588 |
|  | Non-Dominant Handgrip Strength | 0.292 | 0.064 |
|  | Dominant Handgrip Strength | 0.174 | 0.263 |

Note: All correlations are Spearman’s rank correlation coefficients. Only correlations with p < 0.05 are shown in the main Table 4; this supplementary table provides the full matrix including non‑significant correlations.

PUCAI, Pediatric Ulcerative Colitis Activity Index; PGA, Physician Global Assessment; CRP, C-reactive Protein; ESR, Erythrocyte Sedimentation Rate
